# Corrections to traditional methods of verifying tangential‐breast 3D monitor‐unit calculations: Use of an equivalent triangle to estimate effective fields

**DOI:** 10.1120/jacmp.v4i1.2541

**Published:** 2003-01-01

**Authors:** Karl L. Prado, Steven M. Kirsner, Rolly C. Erice

**Affiliations:** ^1^ Department of Radiation Physics The University of Texas M. D. Anderson Cancer Center 1515 Holcombe Boulevard Houston Texas 77030

**Keywords:** breast dose calculations, 3D dosimetry verification, monitor‐unit calculations, loss of scatter corrections

## Abstract

This paper describes an innovative method for correctly estimating the effective field size of tangential‐breast fields. The method uses an “equivalent triangle” to verify intact breast tangential field monitor‐unit settings calculated by a 3D planning system to within 2%. The effects on verification calculations of loss of full scatter due to beam oblique incidence, proximity to field boundaries, and reduced scattering volumes are handled properly. The methodology is validated by comparing calculations performed by the 3D planning system with the respective verification estimates. The accuracy of this technique is established for dose calculations both with and without heterogeneity corrections. © *2003 American College of Medical Physics.*

PACS number(s): 87.53.– j

## INTRODUCTION

The incorporation of a 3D treatment‐planning system and associated monitor‐unit (MU) calculations into clinical practice has challenged traditional MU calculation verification methods. Conventional 2D planning systems and traditional MU calculation verification methods assumed normal beam incidence on a slab of material affording full scatter conditions. This is often called “water phantom geometry.” Inherent in this supposition that full scatter conditions exist is the assumption that irradiated volumes on either side of the calculation plane are identical to the original calculation plane.

Newer planning systems now correctly model three‐dimensional radiation transport, producing more accurate estimates of dose deposition. In many clinical situations, the irradiated 3D volume is significantly different from water‐phantom conditions and fairly large differences emerge between the 3D calculation and traditional 2D verification calculations. This is the case for intact breast tangential field dosimetry.

It has been shown that 3D calculations of breast dose during tangential irradiation can be quite accurate.^1^ The use of the MU settings produced by these 3D calculations is highly desirable. Clinical physics practice standards require that MU settings be verified by an independent calculation. The agreement between the two methods must fall within a specified range (e.g., ±2%). Traditional methods of MU verification failed to routinely fall within this range when needed to verify the tangential‐breast‐field MUs produced by 3D treatment planning systems. Therefore, suitable corrections to the verification calculations were needed before clinical implementation of the 3D calculations.

This paper describes a method to correct for differences between standard verification calculations and 3D‐based breast dose calculations in clinical practice. This verification‐calculation methodology emphasizes a technique for estimating “effective fields” that accurately predicts scatter contributions to the dose delivered in the breast. This effective‐field estimation technique is evaluated by comparison to calculations performed by a commercial 3D treatment‐planning system (ADAC Pinnacle,^3^ ADAC Laboratories, Milpitas, CA) for 60 consecutively selected patients. The effect of incorporation of heterogeneity corrections is also assessed.

## MATERIALS AND METHODS

### A. Dose calculation methodology

The methods employed for verifying calculations are based on water‐phantom data and assume normal incidence and full scatter. An MU calculation equation may take a form such as:

$$MU=\left[\frac{{\left(D_{P}\right)}_{d,SSD+d}}{O_{cal}\times OF_{CS}\times {\left(NPSF\right)}_{CS}^{FS}\times {\left(ISF\right)}_{SSD+d}^{dcal}\times {\left(TMR\right)}_{d,FS}\times {\left(OAF\right)}_{x,d}\times TF\times {\left(WF\right)}_{\theta ,x}}\right].$$

This equation computes the MU needed to deliver dose $D_{P}$ at point *P* located at depth *d* and distance SSD+d. In this equation, $O_{cal}$ is the dose output at the point of calibration, $OF_{CS}$ is the output factor for the collimator setting CS, ${\left(\text{NPSF}\right)}_{\text{CS}}^{\text{FS}}$ is the ratio of the effective (FS) to open field (CS) normalized peak‐scatter factors, ${\left(\text{ISF}\right)}_{SSD+d}^{dcal}$ is the inverse‐square factor that provides the output's distance correction from the calibration distance, $d_{cal}$, to the calculation distance SSD+d, ${\left(\text{TMR}\right)}_{d,FS}$ is the tissue‐maximum ratio for the depth *d*, and (effective) field size (FS), and ${\left(\text{OAF}\right)}_{x,d}$ is an off‐axis factor that accounts for changes in intensity as a function of depth *d* and distance *x* from the central axis. (In this formalism, the distance *x* is defined at isocenter.) The factor TF is the tray transmission factor, and the factor, ${\left(\text{WF}\right)}_{\theta ,x}$ is the $\theta^{{}^{\circ}}-\text{wedge}$ transmission factor. In off‐axis calculation situations, the factor ${\left(\text{WF}\right)}_{\theta ,x}$ represents wedge transmission along the off‐axis ray line a distance *x* from the central ray. (Again, the distance *x* is defined at isocenter.)

Water‐phantom‐based verification methods fail in treatment situations that differ significantly from water phantom conditions. Such is the case of tangential breast irradiation. There is often a reduction of scattered radiation contributing to dose calculation points within the irradiated breast. This reduction can be attributed to many factors. First, calculation points may be located near field boundaries as opposed to the field center. Second, tangential beams are often incident at fairly steep oblique angles rather than at normal incidence. This condition can further reduce the amount of scatter radiation at the dose calculation point. Finally, breast volumes are relatively small compared with full‐scatter geometries and the contour of the breast changes rapidly off the central calculation plane. Therefore, traditional methods of estimating the “effective field” or “equivalent square” of tangential breast fields may not be rigorously suitable.

The factors $O_{cal}$, ${\text{OF}}_{CS}$, ${\left(\text{ISF}\right)}_{SSD+d}^{dcal}$ and TF are all functions of the given irradiation geometry and conditions external to the patient. Proper determination of these factors is normally unambiguous. The factors ${\left(\text{NPSF}\right)}_{CS}^{FS}$ and ${\left(\text{TMR}\right)}_{d,FS}$ and to some degree ${\left(\text{WF}\right)}_{\theta ,x}$, on the other hand, are functions of the “effective field size” of the beam within the patient. Their values depend on the amount of scatter produced within the irradiated volume and contributing to the dose at the calculation point. The estimation of the effective field size of a tangential field is subjective and imprecise. The goal of this study was to devise an explicit and objective method for estimating an “effective field size” that would accurately account for the amount of scatter present in the irradiated breast.

### B. Estimation of effective field size

Breast treatment planning at the University of Texas M.D. Anderson Cancer Center is CT based. Tangential fields used to treat the intact breast are almost always asymmetric. The isocenter of the fields is placed at a stable and reproducible position within the patient and at a point in the area of the chest wall where our clinicians prescribe the dose. As a consequence, the central rays of the beams are commonly located close to the posterior edge of the tangential fields. A beam's eye view of a representative tangential field is shown in Fig. 1. The isocenter in this case is approximately 3 cm from the posterior field edge; however, it is not uncommon for the isocenter to be as close as 1.5 to 2.0 cm from the field edge.

**Figure 1:**
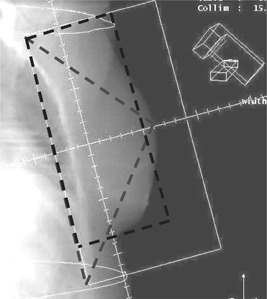
A fairly common medial‐tangential breast field is asymmetric. Its central axis is often 1.5 to 3.0 cm from the medial border of the field. Two methods can be used to estimate the “effective” size of the field. An equivalent square method can assume a rectangular field as shown. The equivalent triangle method assumes that the field's area can be represented by a triangle (also shown) whose base is equal to the length of the field and whose height is equal to the distance from the medial border of the fields to the apex of the breast.

The effective size of tangential fields had been customarily estimated by finding the traditional equivalent square^2^ of the rectangular field. The equivalent square is computed using a field length (*l*) approximately equal to the superior‐inferior field dimension and a width (*w*) equal to the distance from the posterior border of the field to a point approximately 1 to 2 cm inside the apex of the breast (Fig. 1). The field‐width estimate is essentially a field‐size reduction approximation made to eliminate the effect of “flash” or “fall off” outside the breast volume. A “side of the equivalent square” (*s*) is computed using Sterling's equation (2):

$$s=\frac{2lw}{l+w}.$$

The alternative method of estimating the effective field size proposed here utilizes the area of a triangle. The assumption being made is that a triangle is a better approximation to the shape of the scattering volume (a “spherical cap” shape) than is a rectangle. The base (*b*) of the triangle is equal to the length (superior‐inferior) of the tangential field, and its height (*h*) is equal to the distance from the apex of the breast to the medial field border (Fig. 1). The area of this “equivalent triangle” is calculated, and then the square root of the area is then taken to obtain an “effective side” (*s*):

$$s=\sqrt{\frac{h\times b}{2}}.$$

A comparison of calculations performed using these effective‐field estimates to the MU settings calculated using the convolution‐superposition algorithm^3^ of the ADAC Pinnacle^3^ 3D treatment planning system was performed. In all, the treatment plans and calculations of 60 consecutively selected patients receiving intact‐breast tangential irradiation (a total of 120 treatment fields) were assessed by comparing 3D‐system MU settings with the manual verification calculations. In the first 40 of the 60 patients, 3D treatment‐planning system MUs were compared with manual calculations performed using the traditional method and the “equivalent triangle” method of estimating effective fields. All these calculations assumed homogenous media. In the second group of 20 patients, the effects of incorporation of heterogeneity corrections were evaluated by comparing heterogeneity‐corrected 3D treatment‐planning system MU calculations with heterogeneity‐corrected manual calculations performed using the “equivalent triangle” method. This is discussed in more detail in the next section.

**Table I:** Analysis of the frequency distributions of 3D/conventional and 3D/effective‐triangle calculation ratios. 99.7% (3σ) confidence intervals are computed and shown for the mean of each distribution.

	3D/Conventional MU ratios	3D/Triangle MU ratios
*N*=	80	80
Mean ratio	1.022	1.013
Sample standard deviation	0.017	0.011
Uncertainty in the mean	0.0019	0.0013
Upper range (at 99.7% confidence)	1.028	1.016
Lower range (at 99.7% confidence)	1.017	1.009

### C. Heterogeneity effects

Prior to considering incorporation of heterogeneity corrections into breast treatment planning, a “rule” was established that required placement of the dose‐calculation point in breast tissue at a distance of at least 1 to 2 cm from the lung. This would prevent significant differences between homogeneous and heterogeneous plans and calculations. Because verification calculations do not typically consider tissue heterogeneities that may, in fact, be present in the heterogeneity‐corrected 3D plans, a simple correction to the verification calculation was applied when necessary:

$$MU_{c}=MU_{u}\times ({\left(TMR\right)}_{d=ref}/{\left(TMR\right)}_{d=eff}).$$

In the equation shown above, ${\text{MU}}_{c}$ is the MU setting after correction for the presence of heterogeneities, ${\text{MU}}_{u}$ is the uncorrected MU setting, and ${\text{TMR}}_{d=ref}$ and ${\text{TMR}}_{d=eff}$ are the TMRs for the unit‐density (reference) depth and for the heterogeneity‐corrected (effective) depth, respectively. The effective depth accounts for possible differences in CT density within the irradiated volume. It is the physical‐density scaled depth along a rayline from the patient's surface to the calculation point at depth. It is obtained from the 3D treatment‐plan's documentation. In the group of 20 patients used to test heterogeneity‐correction incorporation, triangle‐method verification calculations both with and without TMR corrections were compared to MU calculations resulting from 3D heterogeneity‐corrected treatment plans.

## RESULTS

The calculations of the 80 treatment fields used to treat our initial 40 patients were examined. The results of the comparisons between 3D MU calculations and manual MU verification calculations are analyzed in Tables I and II where the ratios of 3D MUs to conventional MUs are compared to ratios of equivalent‐triangle MUs to 3D MUs. The frequency distributions of these ratio data are shown in Fig. 2.

**Figure 2:**
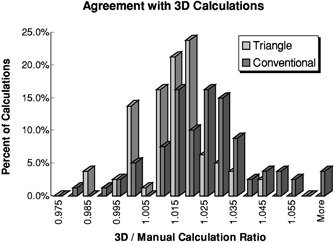
Frequency distribution of the ratios of 3D‐calculated, nonheterogeneity corrected, MU settings to conventional‐based (dark grey) and triangle‐based (light grey) MU homogeneous calculations.

Table I compares the distributions of these ratio data. The mean conventional/3D MU ratio is 1.022±0.006 (at 3σ); the mean triangle/3D MU ratio is 1.013±0.003 (also at 3σ). As shown in this table, in this sample of 80 treatment fields, the means of the two distributions lie within distinctly separate intervals at a 99.7% confidence level. In Table II, a “*z*‐test”^4^ is performed to further validate the difference between the two calculation verification methods. As seen in Table II, the computed *z* statistic for the difference in means lies outside of the 99.9% confidence interval, thus reinforcing the difference between the calculation methods.

**Table II:** Z‐test of the hypothesis regarding the means of the two calculation populations that were sampled. Mean ratios are unequal at a 99.9% confidence level.

Hypothesis	$\mu_{1}=\mu_{2}$
Difference in means	0.0099
Sampling distribution variance	0.0021
“z” statistic	4.7125
Reject hypothesis if outside of:	
@99.0% confidence	±2.576
@99.9% confidence	±3.290

The effect of including of heterogeneity corrections is shown in Table III. The ratios of MU settings calculated using the effective triangle to MU settings calculated by the 3D system are shown. Corrected calculations were obtained from uncorrected calculations by applying the TMR ratio explained above. The mean uncorrected triangle/3D MU‐ratio is 1.006±0.008 (at 3σ), and the mean corrected MU‐ratio is 0.992±0.004 (also at 3σ). Although the mean ratios differ by 1.4%, both verification calculations agree with 3D calculations to within 1% in both uncorrected and corrected settings. As should be expected, however, the precision of the calculations is improved somewhat when heterogeneity corrections are applied given that the 3D treatment plans from this patient group were performed applying heterogeneity corrections.

## DISCUSSION

The results of Tables I and II and the histograms of Fig. 2 suggest that clinical implementation of 3D‐calculated MU settings will result in slightly increased breast dose compared with treatments delivered using conventional MU calculations. In most cases, 3D MUs are 2% to 3% (on the average) greater than traditionally calculated monitor‐unit settings. Some variability in the calculated estimates exists due to the subjective nature of the effective‐field determination.

**Table III:** Effect of incorporation of heterogeneity corrections into the triangle‐method verification calculations. Shown is the analysis of the uncorrected/3D and heterogeneity‐corrected/3D MU ratios. 99.7% 9 (3σ) confidence intervals are computed and shown for the mean of each distribution.

	Uncorrected MU/3D ratios	Corrected MU/3D ratios
*N*=	40	40
Mean ratio	1.006	0.992
Sample standard deviation	0.016	0.008
Uncertainty in the mean	0.003	0.001
Upper range (at 99.7% confidence)	1.014	0.996
Lower range (at 99.7% confidence)	0.998	0.988

3D MU settings can be independently verified more accurately using a better method that accounts for reduced scatter. The equivalent triangle method appears to produce more accurate and precise MU setting estimates. In most instances (almost 90% of the data points), the estimates produced using the equivalent triangle are within 2% of the 3D calculation; about one‐third of them are within 1%. Although it appears that this method continues to overestimate the amount of scatter in the breast (as evidenced by a 3D MU to manually calculated MU ratio that continues to exceed 1.0) by about 1%, the accuracy and precision of this method are clearly superior to previous methods. The coefficient of variation5 of manual calculations using traditional methods is about 2%, whereas that of calculations using the equivalent triangle is approximately 1%.

The fact that the reduction in scatter results from multiple sources requires restatement. Although the proposed equivalent‐triangle effective‐field determination appears to work reasonably well, it does not allow for separation of the different components that contribute to reduction in scatter. The differentiation of scatter reduction due to the presence of field edges or beam obliquity as opposed to scatter reductions produced by decreased breast volume will require additional measurements to properly illustrate.

The presence of heterogeneities does not appear to affect MU verification calculations to an appreciable extent. To a large degree, this may be due to the fact that isocenter (or dose calculation point) placement is restricted to breast tissue and is placed away from significant tissue heterogeneities. Incorporation of heterogeneity corrections routinely (in the form of a TMR ratio) into verification calculations appears to be unnecessary under these circumstances.

The heterogeneity results that are presented here also imply that incorporation of full heterogeneity corrections in 3D treatment planning will not affect the absolute dose delivered to any appreciable extent. The data suggest that the major distinction in absolute dose differences between traditional 2D treatment planning and 3D planning techniques is due to the more accurate modeling of the reduction in scatter rather than to the explicit incorporation of heterogeneity corrections. These results are consistent with those obtained by Ellen *et al.* ^6^ and Pierce *et al.* ^7^

## CONCLUSION

Independent manual calculations that attempt to verify 3D‐calculated intact‐breast, tangential‐field MU settings can be accurately performed using an equivalent triangle method of effective‐field estimation. A review of calculation data encompassing 120 different treatment fields indicates that the effective triangle method can be used to accurately verify 3D MUs to within 2% or better under both homogeneous and heterogeneous conditions.

## References

[1] S.M. Kirsner , K.L. Prado , R.C. Tailor , and J.A. Bencomo , “Verification of the accuracy of 3D calculations of breast dose during tangential irradiation: measurements in a breast phantom,” J. Appl. Clin. Med. Phys. 2, 149–156 (2001). 11602011 10.1120/jacmp.v2i3.2608 PMC5726046

[2] M.J. Day and E.G.A. Aird , “The equivalent field method for dose determinations in rectangular fields,” in “Central Axis Depth Dose Data for Use in Radiotherapy,” Br. J. Radiol. Suppl. No. 17, British Institute of Radiology, London (1983). 6600115

[3] T.R. Mackie , G.H. Olivera , P.J. Reckwerdt , and D.M. Shepard , “Convolution/Superposition Photon Dose Algorithm,” in “General Practice of Radiation Oncology Physics in the 21st Century,” American Association of Physicists in Medicine Monograph No. 26 (Medical Physics Publishing, Madison, 2000).

[4] W.J. Dixon and F.J. Massey , Introduction to Statistical Analysis (McGraw‐Hill, New York, 1969).

[5] I. Miller and J.E. Freund , Probability and Statistics for Engineers (Prentice‐Hall, Englewood Cliffs, NJ, 1965).

[6] M.M. Ellen , K.R. Hogstrom , L.A. Miller , R.C. Erice , and T.A. Buchholz , “A comparison of 18‐MV and 6‐MV treatment plans using 3D dose calculation with and without heterogeneity correction,” Med. Dosim. 24, 287–294 (1999). 10643738 10.1016/s0958-3947(99)00022-9

[7] L.J. Pierce , M.H. Stawderman , K.R. Douglas , and A.S. Lichter , “Conservative surgery and radiotherapy for early‐stage breast cancer using a lung density correction,” Int. J. Radiat. Oncol., Biol., Phys. 39, 921–928 (1997). 9369142 10.1016/s0360-3016(97)00464-1

